# Many Pieces to the Puzzle: A New Holistic Workload Approach to Designing Practice in Sports

**DOI:** 10.1186/s40798-023-00575-7

**Published:** 2023-05-31

**Authors:** Luke Champion, Kane Middleton, Clare MacMahon

**Affiliations:** Sport and Exercise Science (Allied Health, Human Services and Sport), La Trobe University, Melbourne, VIC Australia

**Keywords:** Representative learning design, Practice design, Sports development, Athlete development, Learning transfer, Coaching, Training structure

## Abstract

Representative learning design (RLD) in sport is a well-established concept in both theory and practice. The goal of RLD is to faithfully replicate competition environments in training settings to benefit improvement in athletic performance. There is currently little research that considers how representative an activity needs to be to facilitate learning transfer, and how that level of representativeness might fluctuate between activities or sessions, and across competitive cycles. Similarly, there is no existing research that specifically considers the elevated workload (in cognitive and physical load) of highly representative training, and the potential impacts of chronic overuse of these highly demanding activities. This paper addresses these limitations, making a case for the application of RLD that considers the level of representativeness (fidelity) and the demands placed on athletes (load) from both a cognitive and physical perspective. This paper also suggests several categorisations of training activities that are based on their relative representativeness, level of imposed demands, and the intended outcomes of the activity with reference to the perception–action cycle. The two core concepts of fidelity and load are combined for a new approach to representative training that allows practitioners to balance the benefits of representative training with the risks of imposing excessive load on athletes.

## Key Points

- Representative learning design at present does not adequately prescribe how representative tasks must be to be effective or reflect the broad range of demands placed on athletes.
- Practitioners should consider both representativeness (fidelity) and level of imposed demands (load) when designing training programs for athletes.
- Fidelity and load should be considered from both a cognitive and physical perspective.

## Introduction

How can training be designed to best maximize the benefits to the learner? This is a question that practitioners have been grappling with for a long time, particularly in complex domains with both cognitive and physical demands such as sport. While prior work has provided many situational answers, the nuance of individual circumstances likely means that there is no hard and fast set of rules that dictates what will be most effective for all learners at all times. Different athletes will respond differently to the same training stimulus [1, 2], and the response of each athlete may vary by day or by session due to a range of factors that are out of the practitioner’s control [2]. It is therefore futile to assume that any “one size fits all” approach exists, and instead general principles for training must be adapted to the needs of the individual or group a practitioner is working with to achieve the greatest outcomes. One of these principles of effective training design is ensuring that training considers performance demands and represents the key skills and capabilities that need to be developed in order to progress. The broad line of work dedicated to this idea is known as representative learning design (RLD) [3], and concerns identifying and understanding how elements of the performance environment affect decision making and the performance of skills, and using that understanding to inform the design of training scenarios and learning environments.

The general benefits of representatively designed environments are well established [3, 4]. There is evidence to suggest that highly representative environments produce stronger transfer of skills and knowledge from training to competition [5, 6]. It can also be argued that engaging in periods of physical training at or above a match-like intensity is necessary to ensure that athletes are properly prepared for the rigours of competition [7–9]. There is less consensus, however, on what elements of tasks and environments should be represented and at what stage they should be introduced—both throughout the overall developmental cycle of learners, and within the macro- and micro-cycles of training programs. Research suggests that there are varying needs for task and environmental representativeness as individuals develop expertise [10] and that more variability in practice generally leads to greater performance improvement [11], particularly for individuals who have progressed beyond the initial stages of learning. However, the literature also suggests that training—particularly at the elite level—should be more often than not be as representative as possible [3, 12]. It is likely that there is a place for both high representativeness, while also tailoring representativeness to individual circumstances, such as developmental level and microcycle, as needed. This theoretical optimal blend of training would use a range of different training activities of varying levels of representativeness within the context of a broader training program. To identify this more nuanced approach to representative design across the cycles of learning and practice, it is important to acknowledge the physical and psychological demands that an athlete experiences as a result of varying degrees of representativeness in training, and the implications of those demands for programming of training and recovery.

It is widely acknowledged that performance in competition across a variety of domains is highly demanding and requires recovery [13, 14]; therefore, it is reasonable to expect that fully representative training (i.e. simulated match-play in training) also carries a substantial cognitive and physical load. We know that performing at competition levels of physiological and psychological load over extended periods has detrimental effects on the outcomes experienced by athletes [14]. Specifically, the resulting accumulation of fatigue could lead to injuries or burnout through overtraining [15, 16], at which point any gains are lost. Injuries and burnout occur frequently within athletic populations [17, 18], and as training becomes more representative on a more regular basis, these may become more prevalent. The same volume of training performed with a higher level of representativeness is likely to lead to a greater overall impact on the athlete in many cases, when compared with less representative and more targeted activities. Consequently, the level of demand imposed by training that incorporates highly representative elements, and the subsequent need for recovery and monitoring of cognitive and physical capacity should be considered in the broad design of programs. In order to mitigate the risk of excessive cognitive or physical loading, we propose a new total workload approach to applying representative design principles that considers both the benefits of representativeness and the effect of training demands on the individual. A total workload approach allows practitioners to balance the benefits and drawbacks of highly representative training by taking a holistic view of athlete development and considering a broader range of workload-inducing activities.

For the remainder of the present article, the term load (and workload more generally) is referring to an approximation of the total level of burden or demand imposed on an athlete or group of athletes over a period of training or activity. As a general concept, it can be considered as a product of the amount of training undertaken and the relative intensity of that training (e.g. the cumulative dose of training exposure); however, in specific contexts, it may also refer to something that can be calculated based on some defined parameters (some examples of which will be discussed later in this piece). For example, a high load (or high workload) may refer to a week, session or activity containing either a high volume or high intensity of exercise (or both). Conversely, a low load (or low workload) instead refers to a period of relatively low volume and/or intensity.

### Present Applications of Representative Learning Design

Representative learning design (RLD) aims to ensure that key elements of performance environments are reliably reproduced in practice settings, to facilitate the development of skills and capabilities that readily transfer to performance contexts. Practitioners employing RLD concepts aim to ensure a high degree of representativeness (fidelity) within the training environments they create [3], to make training environments and scenarios feel as close to match-like (or performance-like) as possible for the athlete. This is true both in terms of the amount of cognitive and physical effort that is required to navigate the training scenarios, as well as ensuring that the opportunities for action (affordances) available to learners within those environments are both realistic and equivalent to what would be experienced in competition. For example, a coach may prescribe that the forwards of their football (soccer) team spend a period of time practicing shooting at goal from an approximate distance of 20 m. The representativeness of this task is dependent on a broad range of factors (constraints) that a skilled practitioner is able to manipulate in order to achieve a desired outcome. For this exercise to be highly representative, the coach could incorporate an attacking and defending team, as well as a goalkeeper, in order to maximise the fidelity of the simulated environment. Conversely, if this session consists of repetitions of kicking the ball towards the goal without the presence of additional defenders and a goalkeeper, the exercise is far less representative of the overall skill of shooting for goal in competition. In the latter example, while the motor control patterns and amount of physical effort involved may be similar to a game situation, the relative cognitive effort and level of decision-making are much lower when compared to the more dynamic and less predictable environment presented in the first example. The nuance in the differences between these two variations of the same exercise, and how they replicate or represent different elements will be discussed in greater detail later in this paper. However, they illustrate that representativeness can exist on a spectrum; tasks can be manipulated to allow for increases or decreases in the level of representativeness.

The theory of RLD, while initially applied exclusively to clinical psychology problems [19], has evolved to suit a broad range of applications since its inception in the mid-twentieth century. The use of RLD principles in training for sport (and skilled performance more broadly) is a more modern development [3] and is embedded within several sub-fields and theories such as ecological dynamics [4, 20] and meshed control [21, 22], along with practical methods such as the constraints-based approach to learning design [23]. Ecological dynamics broadly focuses on the interactions between an individual and the environment, and approaches learners as “…complex, neurobiological systems in which inherent self-organization tendencies support the emergence of adaptive behaviours under a range of interacting task and environmental constraints” [4]. Essentially this means that a learner is considered part of a broader ecological system (i.e. environment) that is constantly adapting and evolving; any changes within that system can influence the behaviours of the learner, and vice versa. From this perspective, behaviour is considered as something that emerges from the interaction between the performer and the environment, specifically with the opportunities for action afforded by the environment. Using this perspective, it is asserted that the cycle of perception and action is an expression of cognition, rather than cognition occurring as a central and separate representation that is contained within an anatomical structure (e.g. the brain).

Alternative to ecological dynamics, the theory of meshed control takes many of the same underlying principles (e.g. environment influences the behaviour of the performer) but asserts that there is some degree of central processing of information representing cognition [21, 22]. Rather than direct interaction with the environment and action as cognition without the need for mediating representation, as in ecological dynamics, Christensen and Sutton [22] suggest that movement behaviours are also governed by a higher level of cognition, and that there are both automatic and controlled mediating cognitive processes involved in skilled performance (see Christensen and Sutton [22] for a more detailed explanation).

In practice, the process of action selection within a dynamic system (emergent or otherwise) can be illustrated by considering the interplay between two opposing players. If Player A is in possession of the ball, their actions when approached by Player B will depend on the information they perceive from the environment. Player A will consider their position in relation to both teammates and opponents, and the movements and perceived intention of Player B, when determining their course of action (e.g. pass, dribble, or shoot). Similarly, Player B will use their perception of the environmental information to make their own movements (e.g. tackle, drop to intercept, or hold space). Both players dynamically adapt their behaviour as they receive more information—as other players move, or it starts to rain—and change their actions to suit the new environmental constraints. More successful performers are generally better able to adapt to a broader range of constraints and exploit a greater range of opportunities than their less successful counterparts. It is here that the core difference between the two theories mentioned above is relevant. From a meshed control perspective, this process is inherently driven by higher level cognition but may contain some automatic elements. For example, the decision about what action to take (e.g. dribble or pass) may be cognitive; however, the specific motor processes to carry out that action may be more automatic. From an ecological dynamics perspective, however, the process is instead purely mindless with behaviour simply emerging due to the interactions between various constraints within the environment—the actor instinctively performs an action in response to the challenge they are faced with. While it is important to acknowledge that both views are widely held within sport and skilled performance research, the current paper will primarily explain ideas using a meshed control perspective and with an understanding that cognition plays a key role in skilled performance.

### Ecological Validity in Representative Learning Design

One of the key principles of representative design is understanding that our perceptions affect the decisions we make and the actions we take. Specifically, the environment in which we learn changes both the way we learn, and the way that we are able to apply the learned behaviour later. The concepts of ecological validity and affordances are integral to this understanding. Ecological validity describes how similar the input stimuli are between practice and performance environments (how much it looks and feels like the real thing, e.g. practicing soccer skills on a playing field versus in an indoor gym). Affordances are the opportunities for action that are available to a subject within an environment (e.g. a handle affords grasping, or a defender being out of position affords a shot at goal). A more ecologically valid environment is one where there is a high degree of fidelity between the training stimulus and the intended domain of application, both in terms of affordances, and the sources of information available. In an environment with high ecological validity, the number and range of affordances available to the learner is similar or the same as those they face within competition [24].

The idea of attaining high degrees of ecological validity has inspired work investigating the regularity with which constraints combine to influence behaviour in both competition and training [12, 25, 26]. Robertson et al. [26] used machine learning to identify common constraint combinations that affect kicking in Australian Football match-play. Similarly, Farrow and Robertson [12] mapped the frequency of constraint combinations onto the design and evaluation of training (Skill Acquisition Periodisation framework—SAP). Whilst these approaches are a positive step in the direction of making RLD quantifiable at the elite level, the methods proposed by many of these types of studies remain inaccessible to the majority of practitioners. They require either a high degree of knowledge within the field of ecological dynamics, or access to highly sophisticated technology and programs. In addition, the use of sophisticated methods, such as data analytics and machine learning to make training representative at all times, does not consider the elevated levels of cognitive and physical load experienced by athletes during competition-like training. Other approaches to applying these concepts in practice such as the PoST (Periodisation of Skill Training) framework are more accessible [27]; however, they do not wholly consider or account for the relative demands imposed on the individual in the process.

It is clear there is a need for a conscious effort to determine how much representativeness is needed at different stages of development both within and across competitive seasons, to maximize the learning potential while considering both cognitive and physical workload. While there is clear theoretical evidence in favour of highly representative practice for some part of the whole training program, to our knowledge there has not been any investigation of how much representativeness is needed outside of these full-simulation activities (such as in strength and conditioning training, or similar). There also does not appear to be much consideration of the timing of highly representative training both within typical competitive cycles, and throughout the broader pathway of skill development over a career or lifetime. In addition, there has not been any meaningful attempt to quantify the level of overall cognitive demand placed on athletes by training that is highly representative in nature, or the cognitive workload that they experience in currently unmeasured off-field aspects of their development (e.g. team strategy meetings, preparation for public/media appearances). These gaps in current research and practice lead to the key questions addressed in this paper: How can we ensure optimal learning *and* performance for athletes using principles of representative learning design? How can we do so over both the short- and long-term within a competition cycle, while considering cognitive and physical workload? What are the broader implications of considering cognitive and physical workload within representative design for even longer-term athletic and skilled development?

### Demands of Highly Representative Training

Understanding, quantifying, and manipulating the level of *both* cognitive and physical effort in various degrees of representative training has not yet been investigated in any substantial way. The first step in this investigation is understanding the source of the demands experienced by athletes, by analysing the underlying perception and action process. We must also identify what specific qualities we are aiming to improve through training (with respect to the perception and action process), with the ultimate goal of understanding how different types of training may benefit both individuals and groups of athletes at different stages.

The fundamental basis for Brunswik’s original representative design theory is explained using his Lens model of perception, which describes how he believed humans perceive the world around them [19]. While the Lens model has evolved over time to incorporate other theories [28, 29], it is still largely based on the assumption that humans, in most cases, make probabilistic inferences based on the information they are presented with. That is, they view their environment through a metaphorical lens that transforms raw acoustic and visual information into something that has relevance to their situation [30]. Humans then utilize that probabilistic interpretation of their environment to identify affordances, make decisions, and carry out actions. Those decisions and actions then influence both the state of the environment around them, and the way in which they perceive it, creating what is known as the perception and action cycle [31].

This cycle of simultaneous perception and action is a continuous process that persists as a basic human function not just in sport but across most human endeavours. In our day-to-day life, we are constantly perceiving our surroundings, making decisions, and acting based on our perception of those surroundings, and perceiving how those actions in turn affect or change the environment around us. The fundamental components of perception–action coupling are the uptake of sensory information, the utilization of that information to make decisions, the selection of motor solutions^1^ to address situational needs, and then enacting those motor solutions. This basic process in day-to-day activities is the same across a broad spectrum of situations. When performing more complex and demanding tasks, however, the time limitations, magnitude, and scale of the task for both perception and action are much greater, while the margin for error is far smaller (such as when playing a team sport or driving a manual car on a highway). More demanding settings typically contain more sources of information, shorter time constraints on processing and utilizing that information, a wider range of possible movement solutions but with more specific constraints, and a greater element of risk of injury or task failure when those movements do not go as planned.

The purpose of training is to increase our capacity to carry out each of the sub-processes that form the overall perception and action cycle. That can be by improving our ability to process appropriate information from our surroundings, our ability to make sense of that information to inform decisions, or our ability to recognize affordances and capacity to exploit the movement opportunities available. Representative learning design can be the vehicle that drives this process of developing perceptual and motor capacity, in that the application of those increased capacities to improving performance is greatly enhanced by increased similarity between the training and performance environments [5]. However, that does not necessarily mean that training and competition environments should always be identical. There is evidence to suggest that the transfer of skills or learned behaviours between contexts occurs along a continuum, with greater transfer facilitated by more similar contexts, and less transfer for contexts that differ substantially [32]. It follows that more representative training environments should facilitate greater transfer of skills and learning; however, that does not mean that less representative environments have little or no benefit to an athlete’s performance. Instead, these less performance-like environments (e.g. training in the gym, or using virtual reality), while less representative and consequently less efficient for skill transfer, allow for the targeted manipulation of other performance variables (e.g. strength, speed) and mitigation of specific limitations (e.g. soreness and fatigue) to achieve different outcomes. There is evidence to show that varying levels of representativeness in training change the behaviours observed within otherwise similar activities [33]. Thus, having flexibility in the level of representativeness as needed allows for consideration of the specific goals of training, as well as environmental and individual constraints that a practitioner may not always be able to control, such as weather, injuries, or fatigue.

In a competitive sporting scenario, the amount of perceptual information available to an athlete is immense [34]. This is especially true in team sport, but also applies in any sport where athletes are required to respond to a diverse and dynamic environment. Moreover, the degree of uncertainty involved in tracking and predicting movements and actions of other objects and people within the wider game system is incredibly challenging. The uptake and processing of this information therefore requires a substantial number of cognitive resources from the athlete in order to continuously calibrate and re-calibrate their perception of the environment as it changes over time. Additionally, one must also consider the cognitive effort that is involved in broadly governing the complex movement of the individual as they run, jump, and kick in response to the demands of the task or environment. It has previously been demonstrated that physical demands fluctuate over the course of a competitive team sport season based on a number of factors including (but not limited to) the level of representativeness that is being attained within activities [35]. The cognitive demands imposed on the individual arise from the increased involvement of higher level cognition in making decisions and selecting skills under more restrictive and challenging constraints [21]. Whilst many specific actions may be carried out autonomously, the degree of effort in perception, decision making, and selection of said actions increase as the demands of the task or environment become more complex and less stable—characteristics that are often typical of more representative activities. Given the above, it stands to reason that levels of cognitive demand would also fluctuate heavily in response to variability in training and performance phases, particularly in periods where more representative activities are utilized more frequently. Herein lies the crux of the issue. As we strive to make training and learning tasks more representative of the performance environment in terms of how it constrains and facilitates the emergence of behaviours, we are also increasing the cognitive and physical demands that athletes experience on a regular basis. Cognitive fatigue is known to influence performance in competitive settings [13]. Training at or above competitive levels of demand may lead to an accumulation of both cognitive and physical fatigue, which is not currently considered by any existing models of representative learning design. If the vast scope of previous work that details the importance of adequately monitoring and managing general (physical) load is any indication [14], then it is likely that neglecting to also consider the cognitive equivalent may carry equally dire consequences [16, 36].

## New Considerations for Measuring the Demands of Representative Training

We have established the need for a method of quantifying and planning training with respect to both the benefits to be gained from highly representative design, and the need for a strategic approach to avoid the negative effects of excessive load. To address this balance, any prospective method must consider both the fidelity of training (how realistic it feels to the athlete), and the load experienced, from both a cognitive/psychological and a physical perspective. The combination of these perspectives results in four distinct characteristics that quantify training (Table 1). This method must also account for how the interaction between these four characteristics results in different types of training across typically independent departments and responsibilities within a sport system (e.g. strength and conditioning versus technique training) and whether those types of training address the fundamental goals of improving aspects of the perception and action process (Table 2).

**Table 1:** The four characteristics used to assess training demands

Characteristic	Description
Physical Fidelity	How close to game-like a drill or training session is, physically
Cognitive Fidelity	How close to game-like a drill or training session is, cognitively
Physical Demand	The total physical workload across a drill, session, or cycle of a training plan
Cognitive Demand	The total cognitive workload across a drill, session, or cycle of a training plan

**Table 2:** Intended training goals in reference to the perception action cycle

Perception–action sub-process	Description
Uptake of Information	The capacity to take in (perceive) a range of sensory information
Utilization of Information	The capacity to process and utilize sensory information
Selection of Motor Solutions	The capacity to identify affordances/possible actions
Execution of Motor Solutions	The capacity to successfully carry out actions

**Fig. 1:**
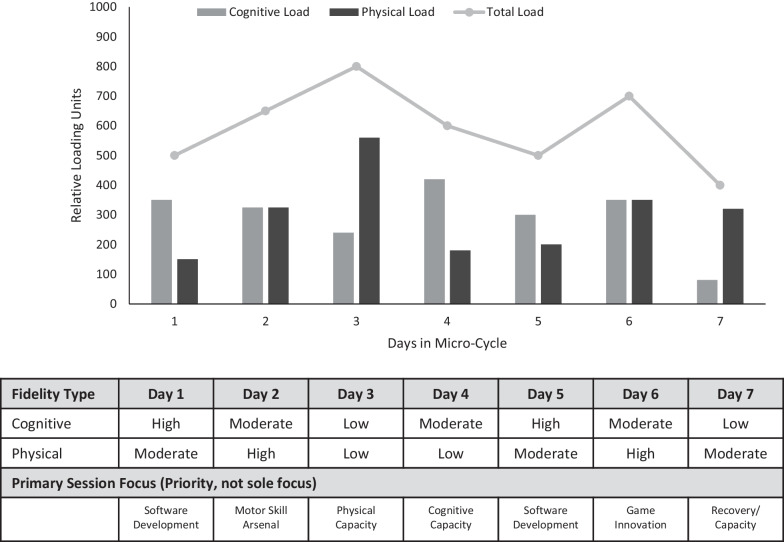
A hypothetical training micro-cycle quantified using the proposed Total Workload Approach to RLD (Session Focus Categories are explained in detail in “Five Types of Training within Total Workload Representative Design” section and Table 4)

These four characteristics (physical and cognitive fidelity, and physical and cognitive demand) (Table1), considered together with training goals within the perception–action cycle (Table 2), can be used to both qualify and quantify different types of training that already exist (these different types of training will be discussed in detail later). Using these descriptive characteristics helps practitioners evaluate the representativeness of drills or training scenarios while also acknowledging the impact that representativeness has on the individual workload of athletes. Using these categories also allows practitioners to plan for sessions of varying levels of representativeness in a targeted manner that provides athletes with adequate time to recover from acute cognitive and/or physical load. This variation in planned activities is possible not just within a session, but also a longer timescale—similar to how physical load is managed now—with planned peaks and troughs in workload that is tapered relative to an athlete or team’s needs at various stages within their training and competitive cycle. For example, within a weekly plan, a team may aim to have a day with a primarily physical focus, a day with a primarily cognitive focus, and other days with roughly comparable levels of cognitive and physical focus (Fig. 1).

Within the hypothetical single week micro-cycle detailed in Fig. 1, we can see that each day has a different level of total workload considering cognitive and physical sources. Each day also has a targeted level of fidelity across both physical and cognitive domains. The interactions between these characteristics dictate what types of training may be appropriate for sessions held on each day. For example, day 3 of the hypothetical week plan has a large physical load, a low cognitive load, and low levels of both physical and cognitive fidelity. These characteristics facilitate some type of gym-based training or equivalent that is not particularly representative of competitive scenarios but does accumulate a substantial level of physical load. In contrast, day 4 has a higher level of cognitive load, lower physical load (to enable recovery from the previous day), a moderate level of cognitive fidelity and a lower level of physical fidelity. A day such as this is well suited to doing primarily off-feet tasks such as video-based training or reviewing footage from previous matches in preparation for upcoming competition periods. There is evidence that video-based interventions and training in tailored virtual environments (e.g. virtual reality or other video-based interventions) may be of benefit when performed with concurrent physical training (in a similar fashion to mental practice), in that the virtual and video-based practice augments the physical practice and is ultimately more effective than physical practice alone [37, 38].

The other day in this hypothetical plan with a substantial disparity between cognitive and physical load and fidelity is day 7. This day is characterised by low levels of cognitive load, moderate to high levels of physical load, and low and moderate levels of cognitive and physical fidelity, respectively. Within a competitive cycle, a session with these characteristics may be a day in which some sort of active recovery type work can be conducted, perhaps after a competitive game or full match simulation the previous day. The fluctuations in both cognitive and physical load that can be seen in this hypothetical plan represent an application of tapering techniques [39] and can be tailored to suit a broad range of needs or competitive settings.

The key intricacies of using this differentiated representativeness method to design and plan training are revealed in the remaining days of the hypothetical plan. Days 1, 2, 5 and 6 have similar levels of both cognitive and physical load, and interchanging levels of fidelity between moderate and high for both cognitive and physical domains. These days would be mostly comprised of more highly representative training and occur at a point within the week where there is time for adequate recovery of cognitive and physical capacities. However, there is the opportunity for even greater specification here, in that these days can further target the development of specific aspects of the perception and action cycle by using different types of training detailed in the following section.

### Relationships Between Cognitive and Physical Load and Fidelity

In the hypothetical training plan in Fig. 1, the relationships between cognitive and physical load and fidelity are complex and have wide ranging implications for the overall impact of training. When descriptive assessments of these qualities are combined, they contribute to the concept of a total workload. That is, the combination of cognitive/physical load and cognitive/physical fidelity can have implications for the total workload of an activity or session. For example, an exercise that is highly physically demanding, but only moderately cognitively demanding, may have an equivalent overall impact on the athlete in workload terms as an exercise that is only moderately physically demanding, but highly cognitively demanding. Similarly, an exercise that is highly representative of competitive scenarios in a physical sense may not be as representative cognitively, but overall may be as representative on average as an exercise where those characteristics are flipped.

**Fig. 2:**
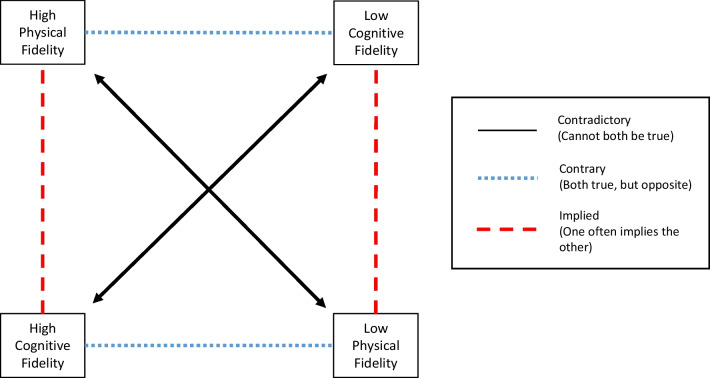
Greimas Square representation of relationships between cognitive and physical fidelity

These relationships between cognitive and physical load and fidelity can be represented by adapting a model known as a Greimas Square (Fig. 2) [40], which allows for a visualisation of how different settings of two different scales interact. The central components of a Greimas Square are two pseudo-axes representing variables that are different but related. A Greimas Square can be used to describe single events, as well as two pairs of lines to indicate contrary and linked settings. The end result is three pairs of variables that are either contradictory, contrary, or often implying the other (Table 3). When cognitive and physical load and fidelity are mapped in this way, it adds context to the second key concept of this paper which is discussed in the next section: the overlapping qualities of different training categories to consider in a total workload approach to representative design.

**Table 3:** The relationships detailed by mapping cognitive and physical fidelity onto a Greimas square

Relationship between characteristics	Logical description	Example pairing
Contradictory	Lie at opposite ends of the same scale and cannot both be true at the same time	High cognitive fidelity and low cognitive fidelity High physical fidelity and low physical fidelity
Contrary	Lie at opposite ends of different scales but can both be true at the same time	High cognitive fidelity and low physical fidelity High physical fidelity and low cognitive fidelity
Implied	Lie at the same end of different scales and there is a general implication that when one is true, the other is also likely to be true	High cognitive fidelity and high physical fidelity Low cognitive fidelity and low physical fidelity

### Five Types of Training Within Total Workload Representative Design

The second key concept of the total workload approach to representative design is the identification of five distinct training categories that are defined by the level of cognitive and physical demands placed on athletes, and the specific perception and action processes targeted (Table 4). It is important to note that presenting these categories is not advocating for the complete decoupling of physical and cognitive demands or training any aspects of performance in complete isolation. Rather, these categories indicate the priorities and goals for a given type of session, with respect to the fidelity requirements and necessary load to achieve those goals. For example, where high physical fidelity is the priority, the tasks used may still require some degree of cognitive effort. Similarly, tasks focused on high cognitive fidelity may still require high physical effort.

**Table 4:** Characteristics of the five proposed training categories

Training category	Physical fidelity	Cognitive fidelity	Physical demand	Cognitive demand	General description of aims	Specific perception–action goals
Fundamental Cognitive Capacity (FC-c)	Low to Moderate	Low to Moderate	Low to Moderate	Moderate to High	Increase capacity to take-in and process sensory information	Uptake of Information
Fundamental Physical Capacity (FC-p)	Low to Moderate	Low to Moderate	Moderate to High	Low to Moderate	Increase capacity to carry out actions through improved flexibility, strength, speed, etc.	Execution of Motor Solutions
Motor Skill Arsenal (MSA)	Moderate to High	Low to Moderate	Low to High	Low to Moderate	Develop the range of actions available to be applied in various scenarios, without specific focus on cognitive elements	Selection of Motor Solutions^a^ Execution of Motor Solutions
Software Development (SD)	Low to Moderate	Moderate to High	Low to Moderate	Moderate to High	Develop the ability to make decisions, but with minimal focus on carrying out actions at competitive levels of performance	Uptake of Information Utilization of Information
Game Innovation (GI)	Moderate to High	Moderate to High	Moderate to High	Moderate to High	Allow exploration of the system of a competition-like environment, identifying affordances and executing on them (Consolidating work from MSA and SD categories)	Uptake of Information Utilization of Information Selection of Motor Solutions^a^ Execution of Motor Solutions
Recovery	Low to Moderate	Low to Moderate	Low to Moderate	Low to Moderate	Regeneration/restoration of cognitive and physical resources and capacities	N/A

^a^”Selection” refers to both deliberate and instinctive actions (see Footnote 1)

**Fig. 3:**
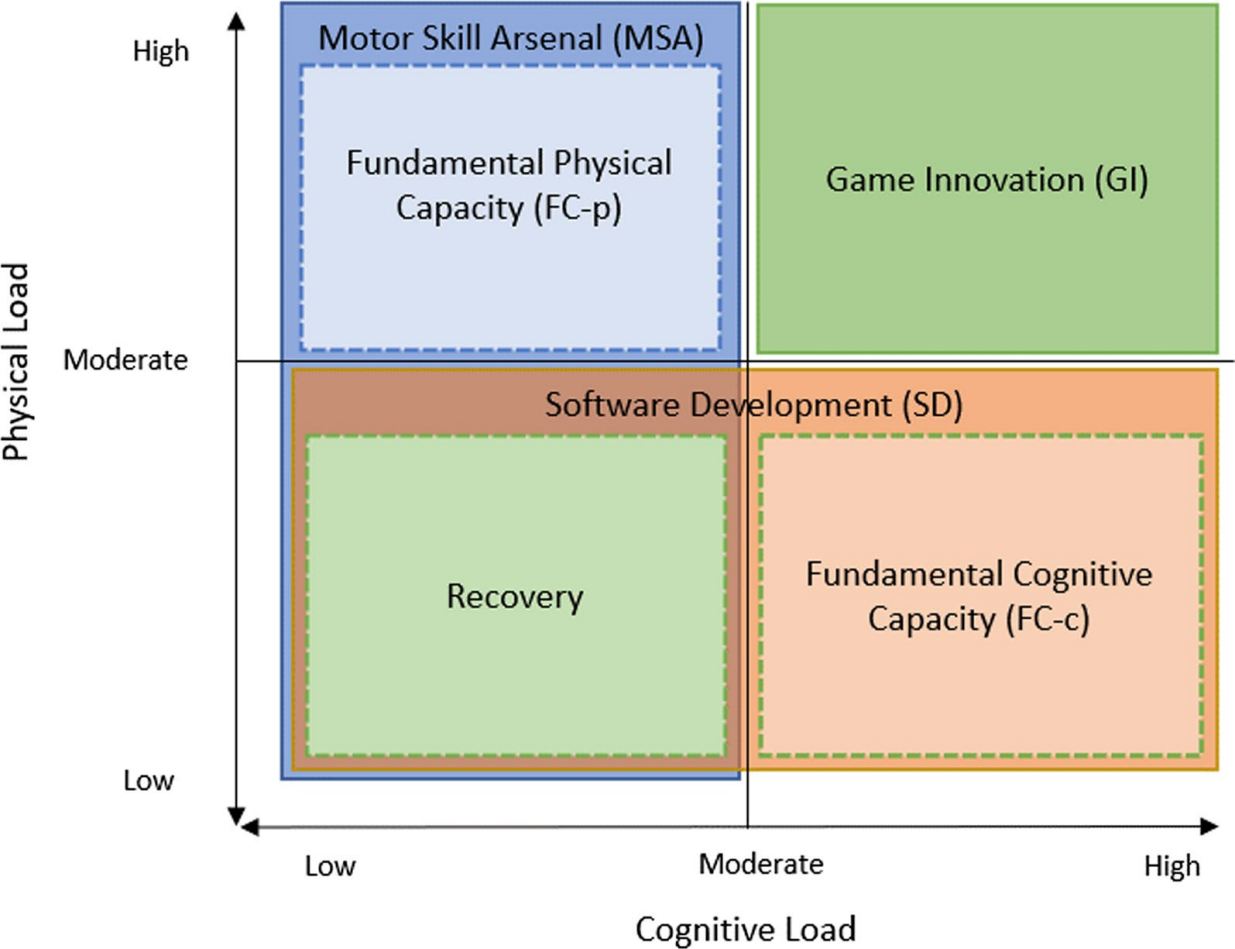
Load properties of the various training categories presented in Table 4. Dashed borders indicate that a category fills the quadrant it is in but has been resized for readability

**Fig. 4:**
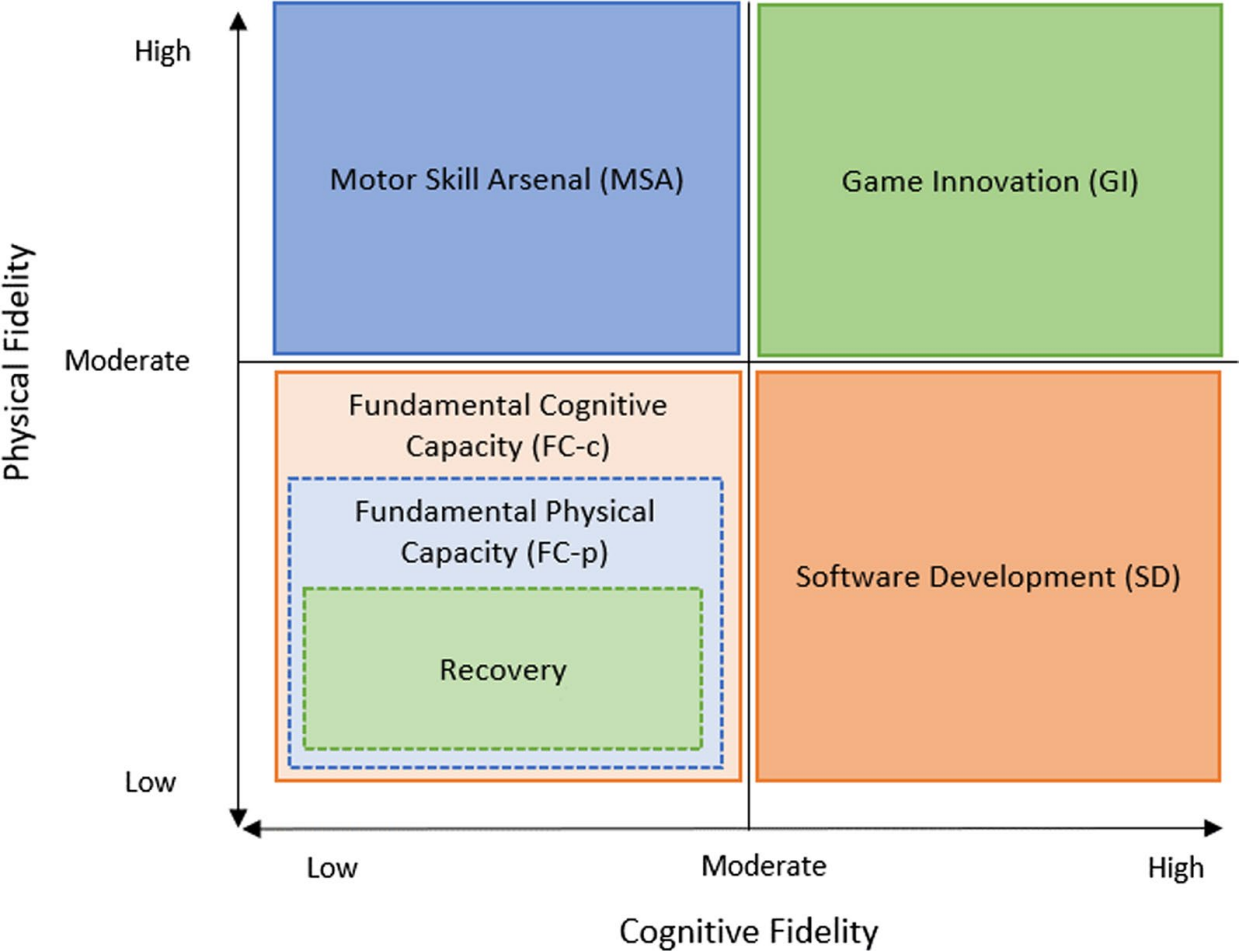
Fidelity properties of the various training categories presented in Table 4. Dashed borders indicate that a category fills the quadrant it is in but has been resized for readability

While the training categories presented in Table 4 are all considered as separate types of activities, it is important to note that there is a great deal of cross-over in qualities that describe each of them. Two activities may have identical load properties, but differing fidelity properties, which allows a practitioner to make an easy distinction between them. However, it is also possible that activities might have overall very similar properties across all qualities, at which point the intended goals of the activity become more relevant. When these categories of training (Table 4) are incorporated into figures that illustrate the load and fidelity properties of various types of training, we can more easily visualise the areas of overlap between these different categories and begin to understand the added importance of considering the training aims/goals in combination with monitoring the execution. This is illustrated by Figs. 3 and 4, which show where each training category would fit when considering physical and cognitive load (Fig. 3) and fidelity (Fig. 4) as perpendicular axes on a pseudo x–y plot.

In Fig. 3, relatively low physical and cognitive demand values could be used to describe as many as three overlapping categories of training (MSA, SD and Recovery). Similarly, a moderate-to-high cognitive demand, and a low-to-moderate physical demand might describe either a FC-c or SD activity classification. When considering both fidelity and level of imposed demand, the only activity that one would expect to exhibit a distinct profile would be the GI category, in that both cognitive and physical demand and fidelity characteristics could be described as moderate-to-high. Where imposed demands have significant crossover between training categories, cognitive and physical fidelity are much more distinct for the majority of categories. High physical fidelity and low cognitive fidelity would most likely indicate a MSA activity, whilst the inverse (low physical, high cognitive) would be indicative of a SD activity. The limited crossover within this series of qualities is seen in the bottom left quadrant of Fig. 4, where activities with low-to-moderate cognitive and physical fidelity could be described as any of FC-c, FC-p or recovery.

The differences between each of these categories of training as illustrated in Figs. 3 and 4 are further clarified by considering the potential types of activities that may be considered within each category. For example, Fundamental Capacity (Cognitive), Software Development, and to some extent Game Innovation training types all may have similar absolute levels of cognitive demand as illustrated in both the table and figure (Table 4, Fig. 3), but where they differ is in either the goals or fidelity of the session (Fig. 4). In this example, a three-dimensional multiple-object tracking (3D-MOT) training activity in virtual reality (FC-c) may be considered as highly cognitively demanding, as may an on field small-sided game (SSG) carried out at walking pace or under specific constraints (e.g. limit on allowed behaviours) (SD), or even a simulated match play activity (GI). However, these are clearly not the same activity, and it is the additional information about the fidelity of these activities that gives greater clarity about the type of training they are (such as whether there is a substantial physical element (SD & GI), and/or whether the aim is to develop capacity to perform or if performance itself is the end goal).

The 3D-MOT (FC-c) example is a somewhat low fidelity task in that it has little in common with a competitive performance in most sports; however, the goal of the training is to increase the capacity to carry out cognitive activity, rather than focussing on the direct application to performance settings. This idea is similar to how resistance training in a gym improves the capacity for physical action but does not typically directly transfer well to skilled performance without additional domain-specific adaptation [41] (see Kalén et al. [42] for a meta-analysis of the importance of domain-specific and domain-general cognitive information in skilled performance). The Software Development example of a SSG at walking pace has somewhat greater cognitive fidelity in relative terms, but still does not overly resemble a competitive setting given that it is a constrained variation on match play. This could be seen as an opportunity to develop domain-specific capacity of cognitive work (i.e. performing skills/making decisions in the correct domain). A walking pace SSG could also be used to provide a reprieve from more highly demanding physical tasks, while still providing some specific cognitive stimulation. In a sense, the walking pace SSG builds upon the fundamental capacity work, as it provides an opportunity to apply improved capacity to track multiple objects (for example), in a more specific setting (theoretically increasing transfer). Finally, the GI example of a full match simulation activity is a task that theoretically approaches maximum fidelity. The intentions of the session are to provide an even more specific environment in which to apply improved capacity, while also consolidating the equivalent physical capacities improved throughout a training program. GI activities are the component of training that brings all other activities together. The stage of a season, or the constraints that an individual is facing (such as fatigue, injury, or even overall skill level) dictate how often GI activities should be performed, and what proportion of training they should make up. GI-type activities are typically the key focus of work within representative design and ecological dynamics; however, there is little research into when and how they should be used. The total workload perspective provides the key considerations for such an examination.

The training categories presented in Table 4, along with the monitoring and periodization of physical and cognitive load and fidelity, demonstrate a new approach to applying RLD that can encapsulate the development of an athlete or team over the full span of their playing career. This approach can be used both in evaluating existing training practices and in planning for future programs. However, we must also acknowledge that this method does not explicitly provide all of the answers that practitioners may require in order to incorporate the principles into their practice. While the intention of this total workload approach is distinctly different from some of the existing approaches (namely SAP and PoST), there is potential for several of these approaches to be used in conjunction given the appropriate resources. Despite the limitations mentioned previously, the SAP framework proposed by Farrow and Robertson provides a robust mechanism for measuring fidelity of performance [12]. Similarly, the PoST framework provides a strong foundation for planned progression of skill development over time and as such could also be utilized [27]. In this example then, one could consider taking the total workload approach presented in this paper as a way of monitoring the use of both of these alternatives. The PoST framework could be used to set overall goals and milestones, the SAP framework in part to quantify fidelity of training, and the holistic workload approach to monitor progression and workload over time.

There are many potential ways to quantify physical load in practice, as evidenced by the great diversity of metrics used in high-performance sport such as high velocity running measurements, algorithmically determined load (such as those calculated by many commercial GPS units), sessional ratings of perceived exertion and others [35, 43, 44]. Similarly, there are several options for defining physical fidelity, such as matching either physical output [45] or the frequency of constraint combinations [12], or even subjective measurement of how game-like a scenario looks and feels to an athlete [46]. The variety of possible measurement highlights the potential flexibility that this total workload approach provides. Programs at the elite level that have access to advanced data analytics methods and a wide variety of performance metrics may be able to incorporate a wide scope of information into the adoption of this approach [35]. Conversely, those at the sub-elite level who may have less capacity to produce and use similar information may be able to use comparatively simpler measurements (e.g. sessional RPE/subjective fidelity ratings) but still achieve beneficial outcomes. Similarly, there are challenges in determining the most appropriate method to measure cognitive load and fidelity.

Monitoring cognitive workload in the manner proposed in this paper is a novel concept, as is fidelity, and as such there are minimal available resources to draw upon when deciding how to quantify it [47, 48]. Whilst there are several subjective rating tools already in use for determining the cognitive workload of tasks in a variety of settings [49], there are no clear purpose-built subjective scales for measuring cognitive workload in sporting environments. It should be noted that this is not a problem unique to sport, with similar issues recognized in other fields involving skilled performance such as performing surgery [50]. Similarly, objective measures of cognitive workload are generally unsuited to the highly dynamic environments presented in sporting scenarios. While tools and techniques such as EEG (electroencephalography), MEG (magnetoencephalography) and pupillometry are all able to provide objective approximations of cognitive workload [51], they also typically require the use of equipment that impairs or limits the ranges of possible movements by the person being monitored. These challenges are not insurmountable; however, it is important to make the case for measuring cognitive workload in this context, in order to guide further development of approaches and technologies to do so. As such, further research within this space is needed to investigate possible measures of both cognitive fidelity and load with respect to planning and monitoring development.

## Conclusion

As our understanding of the factors involved in improving performance grows, we must consider the broad range of demands placed on athletes. As practitioners strive to regularly make training more closely representative of competitive demands, it is critical to acknowledge the variety of demands and the impact they have on the individual. To that end, this paper presents a new approach for practitioners to consider when planning and monitoring representatively designed training. The core principle of this total workload approach is consideration of both the fidelity of training and the imposed demand, from both cognitive and physical sources. Specifically, this approach asks practitioners to consider training activities with respect to the individual adaptations they aim to produce, and to carefully balance the representativeness of training with the need for recovery. This presentation of the total workload approach also defines five categories of training (and their typical characteristics) that may aid practitioners in planning.

The approach outlined in this paper does not explicitly dictate how a practitioner may apply RLD principles, or how they should quantify the four main characteristics of training (cognitive/physical load and fidelity). However, it does act as a platform for future research and future practice, with the intention to bring in to focus the relative demands of various activities that athletes take part in throughout their careers. Specifically, the next key steps are to investigate possible practical applications of this approach and to explore methods of quantifying the demands of representative training across a range of performance domains. This sort of investigation will facilitate deeper analysis of the proposed total workload approach, as well as suggest potential areas in which coaches and practitioners can enhance their current planning strategies.

## Data Availability

Not applicable.

## Footnotes

1 It is important to note here that the process of selecting an action can be either conscious or unconscious (instinctive) and may not always involve explicit decisions. When this process occurs instinctively, the resulting decision or action is more appropriately described as emergent behaviour. The term “selection” in this context throughout this paper is used to refer to both conscious and emergent processes, rather than exclusively one or the other.
